# Waist circumference is a mediator of dietary pattern in Non-alcoholic fatty liver disease

**DOI:** 10.1038/s41598-018-23192-x

**Published:** 2018-03-19

**Authors:** Alireza Ghaemi, Narjes Hosseini, Saeed Osati, Mohammad mehdi Naghizadeh, Azizallah dehghan, Elham Ehrampoush, Behnam Honarvar, Reza Homayounfar

**Affiliations:** 1 Department of Basic Sciences and Nutrition, Health Sciences Research Center, Faculty of public Health, Mazandaran University of Medical Sciences, Sari, Iran; 2 Student Research Committee, Fasa University of Medical Sciences, Fasa, Iran; 3 National Nutrition and Food Technology Research Institute, Faculty of Nutrition Sciences and Food Technology, Shahid Beheshti University of Medical Sciences, Tehran, Iran; 4 Noncommunicable Diseases Research Center, Fasa University of Medical Sciences, Fasa, Iran; 5 Health Policy Research Center, Institute of Health, Shiraz University of Medical Science, Shiraz, Iran

## Abstract

Non-alcoholic fatty liver disease (NAFLD) is an example of pathological fat accumulation in the liver and one of the major health conditions in the world. This study aimed to examine the independent role of dietary patterns in the development of NAFLD. In a cross-sectional study, 1500 individuals referred to a nutrition clinic were randomly selected, their demographic, anthropometric and blood metabolic indices were obtained, and food frequency questionnaires were completed for them. Liver stiffness was calculated using the NAFLD score formula and fibroscan. The two dominant dietary patterns identified were the “healthy” and “unhealthy dietary patterns”. A significant percentage of those with NAFLD (45%) were in the upper quartile of the unhealthy model; however, only 10% had the healthy pattern (p < 0.001). In this study, 32.9 and 13.9% of the healthy and unhealthy participants were in the upper quartile of the healthy diet pattern. Also, it was shown that waist circumference is a strong mediator of dietary patterns and NAFLD relationship, and the indirect effect of diet through abdominal circumference is 28 times greater than the direct effect on NAFLD. The results suggested that healthy and unhealthy dietary patterns are respectively associated with lower- and higher-risk of NAFLD but the role of waist circumference as a mediator deserves more consideration.

## Introduction

Non-alcoholic fatty liver disease (NAFLD) is a type of chronic liver disease characterized by oxidative stress, inflammation and fibrosis in the hepatocytes. It is an example of pathological fat accumulation (mainly triglyceride) in the liver, which shows no symptom of liver disease^1^. Fat constitutes over 5–10% of the liver weight in patients with NAFLD^2^. The disease ranges from simple steatosis to non-alcoholic steatohepatitis and occasionally cirrhosis and hepatocellular carcinoma. It is mainly associated with obesity, dyslipidemia, hypertension, type 2 diabetes and metabolic syndrome^3,4^.

NAFLD is one of the major health conditions worldwide, as 30% of the adult population and 60–80% of diabetic and obese patients are affected by it^5,6^. The prevalence of NAFLD in terms of variables such as age, gender, place of residence and race is between 12–24% in Asia. The overall prevalence of fatty liver in the general Iranian population has been estimated to be 33.9%, a relatively high figure^7^ which reaches 55.6% among those with type 2 diabetes^8^.

The etiology of NAFLD seems to be multicausal. Nutrition has been suggested as a potential environmental factor affecting the risk of NAFLD^9,10^. Recently, considerable attention has been focused on the associations between dietary patterns and the risk of NAFLD. In particular, it has been reported that the Mediterranean diet has a positive impact on NAFLD prevention and treatment^11^.

In recent years, with regards to the relationship between diet and disease, and especially, chronic disease-related diet, researchers investigated the relationships between dietary aspects and health outcomes by analyzing “diet patterns”. With such an analysis, questions on the confounding nutritional factors and the interactions between foods and nutrients were somewhat overcome. On the other hand, dietary patterns reflect the overall dietary intake and thus provide a perspective beyond the effects of nutrients or foods alone because nutrients are not consumed solely and nutrients in different foods can interact with each other or have synergistic effects^12^.

Due to the significance of this issue, the high prevalence of NAFLD and the fact that the onset and development of this disease are closely related to dietary pattern and lifestyle, attempts were made to examine the direct and mediated relationship between dietary patterns and NAFLD by comparing the dietary habits of those with/without NAFLD, in order to take a step towards combating and preventing the disease.

## Results

Of the total sample size of 1500 individuals included in the study, 624 suffered from NAFLD (41.6%) and 876 were healthy (58.4%). Table 1 shows the baseline information of the participants of the study with/without NAFLD. Taking the relatively large sample size into account, all the study variables showed a significant difference between the two groups. No significant difference was observed between the two groups in terms of gender and smoking. The odds ratio for all the variables was calculated for NAFLD with all playing a part in its development.

**Table 1:** Demographic and basic information of the participants in healthy and patient groups.

		Healthy people (N = 876)		NAFLD patient (N = 624)		P-value	OR	95% C.I.	
		Mean	SD	Mean	SD			Lower	Upper
Age (years)		34.09	10.26	57.24	8.69	<0.001	1.243	1.216	1.27
Weight (Kg)		79.88	11.90	98.88	10.05	<0.001	1.148	1.132	1.163
BMI		25.16	3.63	31.73	3.17	<0.001	1.659	1.58	1.743
Waist circumference (cm)		94.90	15.21	120.24	11.35	<0.001	1.133	1.119	1.147
FBS (mg/dL)		97.80	12.36	120.81	11.71	<0.001	1.15	1.135	1.165
Fast Insulin (µU/mL)		7.67	3.30	14.71	3.00	<0.001	1.834	1.73	1.946
Two Hour Glucose (mg/dL)		113.72	18.14	131.55	20.26	<0.001	1.048	1.042	1.055
Two Hour Insulin (µU/mL)		39.05	11.59	55.29	12.15	<0.001	1.116	1.103	1.129
HOMA_IR		0.92	0.23	1.18	0.22	<0.001	143.4	81.70	251.8
HOMA_B		67.77	16.04	79.54	18.55	<0.001	1.04	1.034	1.047
LDL (mg/dL)		92.04	15.00	114.52	15.88	<0.001	1.095	1.085	1.105
HDL (mg/dL)		49.48	7.69	38.44	6.52	<0.001	0.823	0.807	0.839
TG (mg/dL)		185.07	23.84	216.91	23.93	<0.001	1.054	1.048	1.06
Total Cholesterol (mg/dL)		176.59	14.93	196.55	15.62	<0.001	1.086	1.076	1.095
Systolic Blood Pressure(mmHg)		12.41	1.66	13.04	1.66	<0.001	1.256	1.179	1.338
Diastolic Blood Pressure(mmHg)		7.97	1.00	8.92	0.93	<0.001	2.676	2.361	3.033
ALT (IU/L)		38.93	12.66	60.46	12.46	<0.001	1.132	1.119	1.146
AST (IU/L)		30.99	12.94	50.72	11.43	<0.001	1.126	1.113	1.14
GGT (IU/L)		22.85	9.55	37.34	13.04	<0.001	1.117	1.104	1.13
		n	%	n	%				
Sex	Female	534	61.0%	396	63.5%	0.325	1.112	0.900	1.375
Smoking	Yes	107	12.2%	71	11.4%	0.622	0.923	0.671	1.270

ALT, alanine aminotransferase; AST, Aspartate Aminotransferase; BMI, body mass index; FBS, Fasting Blood Sugar; GGT, gamma-glutamyl transferase; HDL, high-density lipoprotein; HOMA-IR, homeostasis model of insulin resistance; HOMA-β, Homeostatic model assessment of β cell function; LDL, low-density lipoprotein; TG, Triglyceride.

In the next step, using factor analysis method, two dominant food patterns were identified for the participants. The foods in each group were labeled “healthy food pattern” and “unhealthy food patterns” (Table 2). The labeling is due to the considerable presence of ready-made, fried and industrial foods in one food pattern and the presence of vegetables, fruits and whole grains in the other. Table 2 shows the score of each food item in both food patterns. For simplification purposes, scores below 0.2 were deleted.

**Table 2:** Load factors of food groups in the two main dietary patterns.

	Unhealthy diet	Healthy diet
Processed meat	0.522	
Mayonnaise	0.510	
Egg	0.452	
Snacks	0.422	
Cereals	0.408	−0.250
Solid. Oils	0.392	
Red Meat	0.389	−0.262
Sweets	0.373	
Carbonated Drinks	0.372	
Fried. Potato	0.366	
Visceral Meat	0.322	
Fruit. Juice	0.293	
Industrial Fruit Juice	0.279	
High. fat. dairy	0.276	
Nuts	0.229	
salt	0.222	
Garlic	0.221	
Legumes	0.221	0.210
sugars	0.210	
seasoning		
Margarine		
Olive		
Pickles		
Liquid. Oils		
Dried fruit		
Other. Vegtables		0.737
Yellow. Vegetables		0.572
Dough		0.489
Low. fat. Dairy		0.479
Vegetables. Cabbages		0.414
Tomato	0.285	0.386
poultry		0.348
coffee		0.326
butter	0.227	−0.268
Potato		0.252
Tea		
fruit		
Whole. wheat. bread		
Fish		
Green.Vegtables		

Coefficients smaller than 0.2 excluded.

Attempts were made to show the relationship between NAFLD and healthy or unhealthy diets (Table 3). Table 3 shows that although a healthy/unhealthy diet has a direct effect on NAFLD, the significance disappeared after optimization, which suggests the probability of strong mediators. A significant percentage of those with fatty liver (45%) were in the upper quartile of the unhealthy model; however, the percentage for the healthy individuals was about 10%, showing a statistically significant difference (P < 0.001). In terms of following a healthy diet, a significant percentage of healthy individuals was in the upper quartile of healthy diet (32.9%); whereas, this figure for those with fatty liver was about 13.9%. Tables 4 and 5 supplementary file shows similar results by FLI Score (43.10% of those with NAFLD was in quartile 4 of unhealthy dietary pattern vs 43.90% of healthy people was in quartile 4 of healthy dietary pattern).

**Table 3:** Prevalence of fatty liver in healthy and unhealthy dietary pattern quartiles (according to NAFLD Score^13^).

		Fatty Liver				Crude				Adjusted^*^			
		No		Yes		P-value	OR	95%CI		P-value	OR	95%CI	
		N	%	N	%			lower	upper			lower	upper
Unhealty Diet	Q1	267	30.5%	108	17.3%								
	Q2	265	30.3%	110	17.6%	0.872	1.026	0.749	1.407	0.111	1.571	0.901	2.739
	Q3	249	28.4%	125	20.0%	0.172	1.241	0.910	1.692	0.524	1.272	0.607	2.664
	Q4	95	10.8%	281	45.0%	<**0.001**	**7.313**	5.296	10.096	**0.015**	**2.730**	1.211	6.155
Healty Diet	Q1	160	18.3%	215	34.5%								
	Q2	208	23.7%	166	26.6%	<**0.001**	**0.594**	0.445	0.793	0.269	0.764	0.473	1.232
	Q3	220	25.1%	156	25.0%	<**0.001**	**0.528**	0.395	0.705	0.463	0.829	0.503	1.367
	Q4	288	32.9%	87	13.9%	<**0.001**	**0.225**	0.164	0.308	0.087	0.614	0.351	1.074

*Adjusted for Age, sex.

**Table 4:** Mediators for unhealthy dietary pattern on fatty liver.

	Indirect effect	Direct effect	Indirect/direct effect	Mediator tests		
				Aroian	Goodman	Sobel
Systolic Blood Pressure(mmHg)	0.02	0.43	0.06	3.27	3.30	3.29
HOMA_B	0.27	0.41	0.65	5.42	5.45	5.43
HOMA_IR	0.01	0.41	0.03	5.48	5.50	5.49
Two Hour Insulin (µU/mL)	0.49	0.37	1.32	7.26	7.28	7.27
GGT (IU/L)	0.47	0.35	1.36	8.41	8.43	8.42
Total Cholesterol (mg/dL)	0.52	0.34	1.54	8.88	8.90	8.89
Two Hour Glucose (mg/dL)	0.35	0.34	1.06	8.95	8.98	8.96
HDL (mg/dL)	0.49	0.34	1.44	9.04	9.06	9.05
Diastolic Blood Pressure(mmHg)	0.07	0.34	0.22	9.05	9.08	9.06
LDL (mg/dL)	0.57	0.28	2.01	10.25	10.28	10.27
TG (mg/dL)	0.54	0.22	2.48	12.11	12.13	12.12
Weight (Kg)	0.64	0.18	3.58	12.65	12.67	12.66
FBS (mg/dL)	0.69	0.18	3.95	12.76	12.78	12.77
Age (years)	0.79	0.13	6.31	13.07	13.09	13.08
AST (IU/L)	0.63	0.15	4.30	13.50	13.52	13.51
ALT (IU/L)	0.67	0.14	4.93	13.51	13.53	13.52
BMI	0.56	0.15	3.73	13.55	13.57	13.56
Fast Insulin (µU/mL)	0.60	0.12	5.09	13.78	13.80	13.79
Waist (cm)	0.79	0.03	28.25	14.64	14.66	14.65

ALT, alanine aminotransferase; AST, Aspartate Aminotransferase; BMI, body mass index; FBS, Fasting Blood Sugar; GGT, gamma-glutamyl transferase; HDL, high-density lipoprotein; HOMA-IR, homeostasis model of insulin resistance; HOMA-β, Homeostatic model assessment of β cell function; LDL, low-density lipoprotein; TG, Triglyceride.

**Table 5:** Mediators for healthy dietary pattern on fatty liver.

	Indirect effect	Direct effect	Indirect/direct effect	Mediator tests		
				Aroian	Goodman	Sobel
Systolic Blood Pressure(mmHg)	−0.02	−0.36	0.07	−4.20	−4.26	−4.23
HOMA_B	−0.27	−0.34	0.78	−5.07	−5.10	−5.08
Diastolic Blood Pressure(mmHg)	−0.05	−0.31	0.17	−6.94	−6.97	−6.95
GGT (IU/L)	−0.47	−0.27	1.74	−7.43	−7.45	−7.44
Two Hour Insulin (µU/mL)	−0.50	−0.28	1.78	−7.52	−7.54	−7.53
HOMA_IR	−0.02	−0.29	0.06	−7.73	−7.75	−7.74
Two Hour Glucose (mg/dL)	−0.39	−0.28	1.38	−8.37	−8.40	−8.39
Total Cholesterol (mg/dL)	−0.53	−0.26	2.02	−8.69	−8.71	−8.70
HDL (mg/dL)	−0.50	−0.25	1.96	−8.78	−8.80	−8.79
LDL (mg/dL)	−0.59	−0.23	2.57	−9.87	−9.90	−9.88
TG (mg/dL)	−0.57	−0.18	3.16	−10.99	−11.01	−11.00
FBS (mg/dL)	−0.71	−0.13	5.35	−11.12	−11.13	−11.12
ALT (IU/L)	−0.67	−0.15	4.32	−11.40	−11.42	−11.41
Weight (Kg)	−0.65	−0.16	4.02	−11.64	−11.66	−11.65
AST (IU/L)	−0.64	−0.14	4.45	−12.00	−12.02	−12.01
Age (years)	−0.80	−0.08	9.71	−12.01	−12.03	−12.02
Fast Insulin (µU/mL)	−0.58	−0.09	6.41	−12.51	−12.53	−12.52
BMI	−0.54	−0.11	4.74	−12.55	−12.57	−12.56
Waist (cm)	−0.75	−0.05	14.34	−12.64	−12.66	−12.65

ALT, alanine aminotransferase; AST, Aspartate Aminotransferase; BMI, body mass index; FBS, Fasting Blood Sugar; GGT, gamma-glutamyl transferase; HDL, high-density lipoprotein; HOMA-IR, homeostasis model of insulin resistance; HOMA-β, Homeostatic model assessment of β cell function; LDL, low-density lipoprotein; TG, Triglyceride.

**Figure 1:**
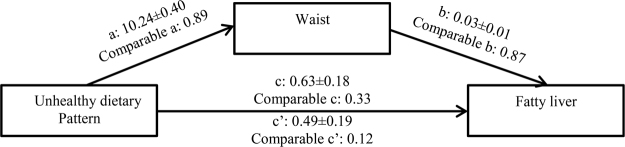
Waist as the most important mediators for unhealthy dietary pattern in fatty liver.

Table 4 was obtained after performing a statistical test and drawing a relationship triangle for each risk factor. This table presents a comparison of the statistic value of the Sobel test and the normal table. If the value is greater than 1.96, a significant mediator exists. The strongest mediator is the one with the greater Sobel statistic. Waist circumference is the strongest mediator. The indirect effect is the effect that a diet has on fatty liver through a mediator. The direct effect is the amount of effect exerted directly on fatty liver irrespective of a mediator. The ratio showed that the indirect diet effect through waist circumference is 28 times greater than the effect exerted on fatty liver directly. The obvious conclusion is that the nullification of this relationship through a mediator is more effective than that of a direct relationship. Waist circumference also showed the strongest relationship in the healthy diet tests (Table 5 and Fig. 1). Adjusting for age and sex showed that indirect effect of unhealthy dietary pattern on fatty liver through waist was 7.2 times more than its direct effect.

A comparison of the quantitative characteristics of the participants among the dominant food patterns quartiles is shown in Tables 6 and 7 supplementary file correlation of each characteristics with healthy and unhealthy dietary pattern have been shown.

## Discussion

The results of the current study showed the prevalence of NAFLD among an urban Iranian sample population, which indicated that 40% of males and 42% of females suffered from the disease. Meanwhile, the study of Moghaddasifar *et al*.^7^, which is a meta-analysis of 23 previous studies in Iran, indicated an onset of about 33.9% among the Iranian population, which is seen more prevalently in males. The higher prevalence of fatty liver in the current study samples may be due to various factors such as the participants’ place of residence (residency in metropolitan areas and the capital of the country) and their greater BMIs, which was about 27.8 ± 4.7, clearly showing that the participants were overweight.

Two popular scales were used to define fatty liver: the Fatty Liver Score^13^ and the FLI Score^14^, which of course, had similar results. The results of the comparison of the two methods are shown in Tables 2 and 3 of the supplementary file. The results of the Fatty Liver Score are shown in the Table 3a,b.

This study compared the nutritional behaviors of a 1500-individual sample with/without NAFLD. The extracted patterns, as the dominant food patterns used in the society, are similar to the food patterns in other studies on the Iranian society^15,16^. The unhealthy diet which manifested in the current study due to the high consumption of items such as processed meat, mayonnaise, eggs, red meat, carbonated beverages and sweets, had a direct and positive relationship with fatty liver disease. The participants in the highest quartile of this food pattern were more likely to develop fatty liver than those in the lowest quartile. With items such as vegetables, yellow vegetables, low-fat dairy, cabbage, tomatoes and chicken meat, the healthy food pattern stood on the opposite side of the previous pattern. This study indicates a negative relationship between fatty liver and healthy food pattern. Those who are in the highest quartile of the healthy diet demonstrated the lowest probability of developing NAFLD when compared with those in the lowest quartile. Various studies that either used the general terms “healthy and unhealthy food patterns” as a combination of foods in a pattern or described some specific food items in this type of pattern and their relationship with fatty liver, reported similar results.

The food pattern in the study of Attica, which included potatoes, red and white meat, and meat products, was associated with increased risk of metabolic syndrome, abdominal obesity and dyslipidemia^17^. By studying four food patterns among the Chinese people, Yang *et al*.^18^ demonstrated that those who consumed the highest amounts of animal source foods had a higher chance of developing fatty livers than those with the lowest levels of consumption (prevalence ratio (PR = 1.354; 95% Cl: 1.063–1.724). However, consumers following the cereal-vegetable pattern had a lower chance of developing fatty liver in the highest quartile when compared with the lowest one (PR = 0.777; 95% Cl:0.618–0.977). In a study on adolescents, Oddy *et al*.^19^ showed that adopting a food pattern known as the “Western Food Pattern” at the age of 14 is more prevalently associated with fatty liver at the age of 17 (odds ratio = 1.59; 95% Cl: 1.17–2.14). Their study emphasized that the use of a healthy diet at the age of 14 may prevent the development of fatty liver at the age of 17 (OR = 0.63; 95% Cl: 0.41–0.96). Jia *et al*.^20^ showed a significant relationship between NAFLD and food pattern in females only, demonstrating 2.19 times greater probability of suffering from fatty liver for those in the highest quartile of the high-carbohydrate intake food pattern (95% Cl: 1.40–3.46).

The healthy diet in the current study which is due to the high consumption of vegetables, low-fat dairy and legumes, is remarkably similar to the Mediterranean diet, which has been shown to have a preventive effect on NAFLD^21–23^. For example, a recent study by Baratta *et al*. showed that the Mediterranean diet reduces the risk of NAFLD (OR: 0.801, P = 0.018)^23^. With regards to the probable mechanism associated with a healthy diet and NAFLD, its effect on improving cardiometabolic profiles (such as reducing TG or blood sugar)^24^ or the antioxidant effects^25^ of diet high in vegetable content, can be pointed to. On the other hand, the recommendations of the American Heart Association, which emphasizes on dietary patterns (such as the DASH or Mediterranean diet) rather than specific food items^26^ can be referred to.

Other studies have discussed the relationship between food intake and fatty liver or its related conditions. In a cross-sectional study, Williams *et al*. reported that a balanced diet accompanied by frequent consumption of raw vegetables, salad, fruit, fish, pasta, rice, and a low consumption of fried foods, sausages, fried fish and potatoes is negatively related to abdominal obesity, glucose, plasma triglyceride and positively related to HDL levels^27^. Recent studies have shown that increased consumption of fruits and vegetables reduces the risk of heart attacks, ischemic heart diseases, hypertension and type 2 diabetes, and contributes to weight loss^28,29^.

There was a significant difference between the affected and unaffected groups in terms of fatty food (such as butter, mayonnaise, high-fat dairy products) and fast food (such as fried potatoes) consumption. Seo *et al*. demonstrated that fast foods have high levels of calorie and fat, thus leading to high intake of energy, fat, salt, inadequate absorption of vitamins and reluctance to consume fruits and vegetables. The high consumption of such foods is associated with weight gain, obesity and/or the risk of chronic diseases such as diabetes^30,31^. Another study on mice showed that fast food diets (with high levels of cholesterol, saturated fat and fructose) were associated with progressive fibrosis^32,33^. Other studies showed that increased fat intake and the Western Diet are associated with insulin resistance, post-meal fat metabolism and progression of NAFLD^34–36^.

This study showed that the consumption of sweet and soft drinks, as well as juices by the affected individuals was higher than the healthy ones. The consumption of sweet drinks is associated with the risk of obesity, metabolic syndrome, fatty liver and heart diseases, which is due to higher calories, as well as rapid and high increase of absorbable sugars^37–39^. These data suggest that high intake of fructose and glucose in the form of sweetened beverages may lead to changes in energy balance regulation at the central nervous system level, or maybe factors other than energy content favor increased calorie consumption and weight gain^2^.

In the current study, the healthy individuals consumed poultry meat more than those with NAFLD. The consumption of fish in the healthy group exceeded that in the unhealthy group. In this regard, it can be asserted from earlier studies that different types of fat might have a protective effect on NAFLD, and the most important one is omega-3 fat^40^. Other laboratory studies have shown that a diet rich in omega-3 fatty acids increases insulin sensitivity in rats, reduces triglyceride contents in the liver and improves fatty liver^41,42^.

In this study, the consumption of cereals by individuals with fatty liver exceeded that of healthy individuals. In a case-control study in China, Shi *et al*. reported no significant difference between the two groups in terms of intake of cereals, carbohydrates and fish in the two groups. However, the intake of fruits, vegetables and dairies in the case group was lower than that in the control group and meat intake in the case group exceeded that in the control group^43^.

It is noteworthy that the affected individuals used natural juices more than the healthy ones. Regarding NAFLD, studies on rodents and humans have shown that high fructose-rich diets result to liver steatosis^44^ and it has been reported that simple carbohydrates such as fructose increase lipogenesis in liver and prevent the oxidation of lipids^45^.

Finally, this hypothesis was examined to determine whether the relationship between food patterns and fatty liver is a direct one or if it interacts with other variables. Tables 4 and 5 show that waist circumference is a strong mediator in the relationship between food patterns and fatty liver. Numerous studies have discussed the relationship between waist circumference and the risk of developing fatty liver. In a meta-analysis study, Qing Pang *et al*.^46^ examined 20 well-structured studies and concluded that waist circumference is the strongest anthropometric variable in predicting fatty liver (OR = 3.14, 95%Cl: 2.07–4.77), an even stronger variable than BMI. There are evidences that insulin resistance in NAFLD is more related to waist girth than BMI, and fatty liver is a liver manifestation of metabolic syndrome^46–48^.

To the best of the researchers’ knowledge, no other study has considered waist circumference as a mediator of the relationship between food pattern and fatty liver in such a clear and strong relationship. It can be assumed that food patterns affect fatty liver by changing waist circumference size, which is an indication of abdominal fat accumulation. The relationship power indicates that keeping waist circumference in a normal range may prevent fatty liver, even if a proper diet is not followed.

The limitations of this study include lack of a biopsy, the most accurate method of assessing the extent of liver damage in NAFLD, which could not be performed on the outpatients, as well as the cross-sectional nature of the study which prevented it from showing causal relationships, and/or even reverse relationships.

In general, the research results suggested that a healthy diet is associated with a lower risk and an unhealthy diet pattern is associated with a higher risk of developing fatty liver. In case the study results are confirmed in prospective studies, the healthy dietary pattern can be used for the development of suitable training policies to promote nutritional awareness, to encourage individuals to follow a healthier dietary pattern and avoid following unhealthy dietary patterns, to control and prevent the development of fatty liver. Abdominal obesity should be considered as a strong mediator affecting dietary patterns and fatty liver, and international recommendations on reducing abdominal obesity should be taken seriously. It is hoped that some steps are taken to prevent NAFLD with more education on the significance of observing a healthy diet plan and reducing abdominal obesity.

## Methodology

The study protocol was in accordance with the Declaration of Helsinki guidelines and was approved by the Institutional Review Board (IRB) of Fasa University of Medical Sciences (Code: IR.FUMS.REC.1396.230). Written informed consent was obtained from all the participants. It was confirmed that all the methods were performed in accordance with the relevant guidelines and regulations.

This cross-sectional study was conducted on 1500 individuals living in Tehran between April 1, 2016 and the end of February, 2017. The participants were selected using random cluster sampling among those visiting a nutrition counseling center in Tehran. The exclusion criteria included alcohol consumption >40 g/day for males, >20 g/day for females, and lack of informed consent. The Ethics Committee of Fasa University of Medical Sciences approved the research protocol with No. 96061. The purpose of the study was explained to the participants and they completed written consent forms before entering the study.

Demographic information including gender, age and menopausal status were obtained. The participants’ heights and weights were measured and recorded by a trained individual. The heights were measured using a stadiometer with a 1.0 cm precision while the weights were determined using a digital scale with a 1.0 kg precision (Seca 767, Japan). The body mass index (BMI) was calculated by dividing weight in kilograms by the square of height in meters.

Blood pressure was measured twice in a sitting position from the right arm after 15 min of rest and the mean of the two figures was considered as the final blood pressure.

The common dietary intake of the participants was obtained using a semi-quantitative food frequency questionnaire (FFQ) over the past year. The questionnaire included a list of 168 food items with a standard amount of each item specified. The validity and reliability of this questionnaire have been previously investigated^49^. The participants expressed the frequency with which they consumed each item with respect to the standard amount in the past year. The values specified for each item were converted to grams per day using the manual for household measures. A comparison of the risk factors in individuals with/without fatty livers was done using the Chi-square test and was demonstrated by the odds ratio and a 95% confidence interval. Two main components of the diet were extracted using principal component analysis (PCA) and varymax rotations.

This method is determined by weight for each of the categories of food in the specified component, which indicates the presence of that food in the target component, and by this, the foods that make up the particular component (factor) is understood and based on the presence of these foods, the component can be named.

The components were labeled “healthy food pattern” and “unhealthy food pattern” in terms of nutrients. Food patterns were categorized into four quartiles, compared using logistic regression in those affected and unaffected, and demonstrated by the odds ratio and a 95% confidence interval. The analysis was performed once using raw data and again after age and gender optimization. The relationship between the two main components of food pattern and risk factors was shown using correlation and regression coefficients.

Variables might play a role, and associated risk factors as the mediator of the role of diet were examined. The mediator is an intermediate variable responsible for the relationship between two other variables.

The following should be considered when examining a mediator:

A. Is the independent variable relates to the mediator? Route a
B. Is the mediator relates to the dependent variable? Route b
C. Is the independent variable relates to the dependent variable? Route c
D. Does the simultaneous examination of the relationship between the independent variable and mediator with the dependent variable reveal the extent to which the relationship between the independent and the dependent variable decreases? Route c’

If Route c’ is adequately smaller than Route c, the mediator has been found.

It is necessary for the mediator to have a biological justification as well. Due to the dichotomous state of the dependent variable, the correction proposed by Nathaniel R^50^ based on the Sobel test^51^ was used to find the mediators. The strongest mediator was determined based on the greater value of the Sobel statistic and was optimized in terms of age and gender.

The i-ntravenous blood sample was taken in a 12-h fasting state to measure the condition of serum lipids. Blood samples were transferred to a laboratory for biochemical testing. Blood sugar (FBS), high-density lipoprotein (HDL), triacylglycerol (TG), total cholesterol (TC) and liver enzymes (ALT, AST and GGT) were measured by an enzymatic method using Pars Azmoon Commercial kits. The Friedewald formula was used to calculate low-density lipoprotein (LDL)^52^. The serum lipid values were reported based on mg/dl.

The homeostatic model (HOMA) is a method used to determine and quantify insulin resistance and beta cell function (equations 1 and 2). IR is used for insulin resistance and %β for the β-cell function.

1 $$\mathrm{HOMA} \mbox{-} \mathrm{IR}=\frac{Glucose\,(\frac{mg}{dL})\ast Insulin\,(\frac{mU}{mL})}{405}$$

2 $$\mathrm{HOMA} \mbox{-} {\rm{\beta }}=\frac{360\,\ast \,Insulin\,(\frac{mU}{mL})}{Glucose\,(\frac{mg}{dL})-63} \% $$

Liver stiffness was measured by the NAFLD Score formula (equation 3) which was used to determine the extent of liver fat^13^ and confirmed by an experienced specialist using a fibroscan device.

3 $$\begin{array}{rcl}{\rm{NAFLDScore}} & = & -\,1.675+(0.037\,\ast \,{\rm{age}}[{\rm{years}}])+(0.094\,\ast \,{\rm{BMI}}[{\mathrm{kg}/m}^{2}])\\  &  & +\,(1.13\,\ast \,\mathrm{IFG}/\mathrm{diabetes}[{\rm{yes}}=1,\,{\rm{no}}=0])+(0.99\,\ast \,\mathrm{AST}/\mathrm{ALT}\,{\rm{ratio}})\\  &  & -\,(0.013\,\ast \,{\rm{platelet}}\,{\rm{count}}[\times 109/L])\,\mbox{--}\,(0.66\,\ast \,{\rm{albumin}}[g/\mathrm{dl}])\end{array}$$

Another powerful indicator of fatty liver diagnosis is the fatty liver index (FLI) which is calculated using the equation 4^14^.

4 $$\begin{array}{rcl}{\rm{FLI}} & = & ({{\rm{e}}}^{{0.953}^{\ast }loge(triglycerides)+{0.139}^{\ast }BMI+{0.718}^{\ast }loge(ggt)+{0.053}^{\ast }waistcircumference-15.745})\\  &  & /(1+{{\rm{e}}}^{{0.953}^{\ast }loge(triglycerides)+{0.139}^{\ast }BMI+{0.718}^{\ast }loge(ggt)+{0.053}^{\ast }waistcircumference-15.745})\,\ast \,100\end{array}$$

The data were analyzed in SPSS ver. 23. The descriptive data for the main variables were reported as mean ± standard deviation. The independent t-test was used to compare the variables between the two groups. Linear regression test was used to determine the linear relationship of the variables used in the study. P < 0.05 was considered as the significance level.

### Implication for health policy makers/practice/research/medical education

Determining the relationship between dietary pattern and other risk factors with NAFLD can help policy makers or other researchers for designing preventive programs for Fatty liver that accounts for 1.3–2.6 deaths per 100,000 per year. The results of present study shows dietary pattern is significant determinant of NAFLD but waist circumference is an mediator of its effect on NAFLD that must be under more control.

## Electronic supplementary material

Supplementary Information
