# Supplementary material for: Waist circumference is a mediator of dietary pattern in Non-alcoholic fatty liver disease
Source: Sci Rep. 2018 Mar 19;8:4788. doi: 10.1038/s41598-018-23192-x (PMC5859081; doi:10.1038/s41598-018-23192-x)
Supplement: Supplementary file 1 — Supplementary Information [file 41598_2018_23192_MOESM1_ESM.doc]

Supplementary Info:

Waist circumference is a mediator of dietary pattern in Non-alcoholic fatty liver disease

**Alireza Ghaemi^1^, Narjes Hosseini^2^, Saeed Osati^3^, Mohammad mehdi Naghizadeh^4^, Azizallah dehghan^4^, Elham Ehrampoush^4,5^, Behnam Honarvar^5^, Reza Homayounfar^4,*,5^**

1. Department of Basic Sciences and Nutrition, Health Sciences Research Center, Faculty of public Health, Mazandaran University of Medical Sciences, Sari, Iran

2. Student Research Committee, Fasa University of Medical Sciences, Fasa, Iran

3. National Nutrition and Food Technology Research Institute, Faculty of Nutrition Sciences and Food Technology, Shahid Beheshti University of Medical Sciences, Tehran, Iran.

4. Noncommunicable Diseases Research Center, Fasa University of Medical Sciences, Fasa, Iran

5. Health Policy Research Center, Institute of Health, Shiraz University of Medical Science, Shiraz, Iran.

Another powerful indicator of fatty liver diagnosis is the fatty liver index (FLI) index, which is calculated using the following formula^1^.

FLI = (e 0.953*loge (triglycerides) + 0.139*BMI + 0.718*loge (ggt) + 0.053*waist circumference - 15.745) / (1 + e 0.953*loge (triglycerides) + 0.139*BMI + 0.718*loge (ggt) + 0.053*waist circumference - 15.745) * 100

Results are also repeated using FLI index, which can be seen in the following tables.

Table 1. Mean and standard deviation, as well as the percentage of people involved in the fatty liver with a FLI index

|  | | Mean | Standard Deviation | |
| --- | --- | --- | --- | --- |
| Fatty Liver Index | | 49.91 | 22.37 | |
|  | | Count | Column N % |  |
| FLI_Cat | Healthy | 376 | 25.1% |  |
|  | Marginal | 479 | 31.9% |  |
|  | Fatty liver | 645 | 43.0% |  |

FLI < 30 categorized as healthy and a FLI ≥ 60 categorized in hepatic steatosis

Table 2. Compare the two FLI and NAFLD score

|  | | Fatty Liver | | | |
| --- | --- | --- | --- | --- | --- |
|  |  | No | | Yes | |
|  |  | Count | Column N % | Count | Column N % |
| FLI Cat | Healthy | 376 | 42.9% | 0 | 0.0% |
|  | Marginal | 441 | 50.3% | 38 | 6.1% |
|  | Fatty liver | 59 | 6.7% | 586 | 93.9% |

The analyzes were carried out to determine the equality of FLI method and our method, and the results indicate a remarkable match between the two methods (Table 2 , 3). In this table, 94% of those who were involved with fatty liver according to the previous index were diagnosed with fatty liver according to the new index. But 50% of those who were healthy based on the index were based on the new indicator in the marginal group. Thus the new index (FLI) has the sensitivity of the previous index (Angulo method), but it has more specificity.

Table 3. Matching two methods of NAFLD diagnosis (FLI and NAFLD score)

|  | | Fatty Liver Score^2^ | | | | | | | |
| --- | --- | --- | --- | --- | --- | --- | --- | --- | --- |
|  |  | 0 | | 1 | | 2 | | 3 | |
|  |  | Count | % | Count | % | Count | % | Count | % |
| FLI Score Categories^1^ | Healthy | 333 | 98.5% | 43 | 8.0% | 0 | 0.0% | 0 | 0.0% |
|  | Marginal | 5 | 1.5% | 436 | 81.0% | 38 | 10.9% | 0 | 0.0% |
|  | Fatty liver | 0 | 0.0% | 59 | 11.0% | 310 | 89.1% | 276 | 100.0% |

FLI < 30 categorized as healthy and a FLI ≥ 60 categorized in hepatic steatosis

Table 4. Demographic and basic information of the subjects participating in the study in healthy and patient groups (according to FLI)

|  | | Healthy people  (N=376) | | Fatty liver  patient  (N=645) | | P-value | OR | 95% C.I. | |
| --- | --- | --- | --- | --- | --- | --- | --- | --- | --- |
|  |  | Mean | SD | Mean | SD |  |  | Lower | Upper |
| Age (years) | | 27.6 | 7.95 | 56.39 | 9.2 | 0 | 1.316 | 1.268 | 1.366 |
| Weight (Kg) | | 73.17 | 9.2 | 98.13 | 10.43 | 0 | 1.225 | 1.196 | 1.254 |
| BMI | | 23.07 | 2.89 | 31.46 | 3.36 | 0 | 2.004 | 1.84 | 2.182 |
| Waist (cm) | | 86.24 | 12.08 | 119.38 | 11.85 | 0 | 1.191 | 1.166 | 1.217 |
| FBS (mg/dL) | | 91.58 | 10.34 | 120 | 11.92 | 0 | 1.213 | 1.185 | 1.241 |
| Fast Insulin (μU/mL) | | 5.72 | 2.58 | 14.41 | 3.2 | 0 | 2.187 | 1.981 | 2.415 |
| 2h Glucose (mg/dL) | | 107.41 | 14.94 | 130.51 | 20.52 | 0 | 1.073 | 1.063 | 1.084 |
| 2h Insulin (µU/mL) | | 34.51 | 10.34 | 54.7 | 12.53 | 0 | 1.148 | 1.129 | 1.167 |
| HOMA_IR | | 0.84 | 0.19 | 1.17 | 0.23 | 0 | 110.648 | 46.019 | 266.042 |
| HOMA_B | | 64.62 | 13.97 | 79.36 | 18.12 | 0 | 1.055 | 1.046 | 1.065 |
| LDL (mg/dL) | | 85.96 | 12.49 | 113.79 | 16.1 | 0 | 1.136 | 1.119 | 1.154 |
| HDL (mg/dL) | | 52.88 | 6.65 | 38.72 | 6.62 | 0 | 0.756 | 0.732 | 0.781 |
| TG (mg/dL) | | 176.02 | 20.53 | 215.35 | 24.41 | 0 | 1.075 | 1.066 | 1.085 |
| Total Cholesterol (mg/dL) | | 170.67 | 12.67 | 195.96 | 15.95 | 0 | 1.124 | 1.108 | 1.14 |
| Systolic Blood Pressure(mmHg) | | 12.09 | 1.54 | 12.97 | 1.69 | 0 | 1.389 | 1.279 | 1.508 |
| Diastolic Blood Pressure(mmHg) | | 7.68 | 0.92 | 8.87 | 0.97 | 0 | 3.596 | 3.023 | 4.277 |
| ALT (IU/L) | | 32.7 | 10.38 | 59.4 | 12.86 | 0 | 1.184 | 1.16 | 1.208 |
| AST (IU/L) | | 24.52 | 11.13 | 49.87 | 12 | 0 | 1.18 | 1.157 | 1.203 |
| GGT (IU/L) | | 20.09 | 8.54 | 36.78 | 12.83 | 0 | 1.15 | 1.129 | 1.171 |
|  |  | n | % | n | % |  |  |  |  |
| Sex | Female | 206 | 54.80% | 400 | 62.00% | 0.023 | 1.347 | 1.041 | 1.774 |
| Smoking | Yes | 49 | 13.00% | 78 | 12.10% | 0.661 | 1.089 | 0.743 | 1.597 |

ALT, alanine aminotransferase; AST, Aspartate Aminotransferase; BMI, body mass index; FBS, Fasting Blood Sugar; GGT, gamma-glutamyl transferase; HDL, high-density lipoprotein; HOMA-IR, homeostasis model of insulin resistance; HOMA-β, Homeostatic model assessment of β cell function; LDL, low-density lipoprotein; TG, Triglyceride.

Table 5- Prevalence of fatty liver in healthy and unhealthy dietary pattern quartiles (according to FLI Score^14^)

|  | | Fatty Liver | | | | Crude | | | | Adjusted^*^ | | | |
| --- | --- | --- | --- | --- | --- | --- | --- | --- | --- | --- | --- | --- | --- |
|  |  | No | | Yes | | P-value | OR | 95%CI | | P-value | OR | 95%CI | |
|  |  | N | % | N | % |  |  | lower | upper |  |  | lower | upper |
| Unhealty Diet | Q1 | 151 | 40.20% | 120 | 18.60% |  |  |  |  |  |  |  |  |
|  | Q2 | 125 | 33.20% | 112 | 17.40% | 0.090 | 1.360 | 0.953 | 1.941 | **0.023** | 2.346 | 1.125 | 4.891 |
|  | Q3 | 66 | 17.60% | 135 | 20.90% | **<0.001** | 2.196 | 1.513 | 3.186 | 0.477 | 1.351 | 0.589 | 3.096 |
|  | Q4 | 34 | 9.00% | 278 | 43.10% | **<0.001** | 10.415 | 6.960 | 15.587 | **0.008** | 8.098 | 1.731 | 37.886 |
| Healty Diet | Q1 | 59 | 15.70% | 214 | 33.20% |  |  |  |  |  |  |  |  |
|  | Q2 | 81 | 21.50% | 169 | 26.20% | **0.005** | 0.583 | 0.402 | 0.846 | 0.693 | 1.148 | 0.578 | 2.281 |
|  | Q3 | 71 | 18.90% | 166 | 25.70% | **0.002** | 0.554 | 0.380 | 0.807 | 0.570 | 0.814 | 0.400 | 1.657 |
|  | Q4 | 165 | 43.90% | 96 | 14.90% | **<0.001** | 0.164 | 0.113 | 0.239 | 0.094 | 0.524 | 0.246 | 1.115 |

*. adjusted for Age,sex,

Table 6. Comparison of the quantitative characteristics of the participants among the dominant food patterns quartiles

|  | Unhealthy Diet | | | | | | | | |  | | Healthy Diet | | | | | | | | |  |
| --- | --- | --- | --- | --- | --- | --- | --- | --- | --- | --- | --- | --- | --- | --- | --- | --- | --- | --- | --- | --- | --- |
|  | Q1 (n=375) | | | Q2,Q3 (n=749) | | | Q4 (n=376) | | | P.value | | Q1 (n=375) | | | Q2,Q3 (n=750) | | | Q4 (n=375) | | | P.value |
|  | Mean | SD | Mean | | SD | Mean | | SD |  | | Mean | | SD | Mean | | SD | Mean | | SD |  | |
| Age (years) | 40 | 15 | 40 | | 14 | 54 | | 13 | <0.001 | | 48 | | 15 | 45 | | 14 | 37 | | 14 | <0.001 | |
| Weight (Kg) | 83.18 | 14.04 | 85.45 | | 13.76 | 97.01 | | 12.54 | <0.001 | | 92.12 | | 14.81 | 88.76 | | 13.52 | 81.47 | | 14.32 | <0.001 | |
| BMI | 26.32 | 4.53 | 27.02 | | 4.26 | 31.19 | | 4.23 | <0.001 | | 29.54 | | 4.59 | 28.17 | | 4.32 | 25.68 | | 4.85 | <0.001 | |
| Waist circumference (cm) | 97.32 | 14.89 | 101.46 | | 16.84 | 121.45 | | 15.39 | <0.001 | | 113.24 | | 19.05 | 105.77 | | 16.24 | 96.98 | | 18.91 | <0.001 | |
| FBS (mg/dL) | 102.92 | 15.49 | 104.23 | | 15.68 | 118.07 | | 14.67 | <0.001 | | 112.75 | | 16.75 | 108.00 | | 15.57 | 100.73 | | 16.19 | <0.001 | |
| Fast Insulin (μU/mL) | 9.19 | 4.45 | 9.61 | | 4.19 | 13.98 | | 4.27 | <0.001 | | 12.24 | | 4.85 | 10.89 | | 4.20 | 8.37 | | 4.68 | <0.001 | |
| 2h Glucose (mg/dL) | 112.99 | 19.09 | 120.51 | | 20.48 | 130.50 | | 20.09 | <0.001 | | 126.99 | | 20.49 | 121.53 | | 21.00 | 114.51 | | 19.53 | <0.001 | |
| 2h Insulin (µU/mL) | 45.19 | 14.42 | 43.59 | | 14.22 | 50.83 | | 12.98 | <0.001 | | 47.82 | | 14.45 | 46.95 | | 14.23 | 41.51 | | 13.33 | <0.001 | |
| HOMA_IR | 1.04 | .28 | .98 | | .25 | 1.11 | | .23 | <0.001 | | 1.06 | | .25 | 1.05 | | .26 | .94 | | .24 | <0.001 | |
| HOMA_B | 70.17 | 16.72 | 71.58 | | 17.67 | 77.32 | | 19.37 | <0.001 | | 75.19 | | 18.88 | 73.12 | | 17.67 | 69.24 | | 17.60 | <0.001 | |
| LDL (mg/dL) | 97.82 | 18.44 | 98.12 | | 17.79 | 111.47 | | 18.14 | <0.001 | | 106.00 | | 18.61 | 102.72 | | 18.41 | 94.13 | | 18.35 | <0.001 | |
| HDL (mg/dL) | 45.23 | 9.52 | 46.67 | | 8.58 | 40.98 | | 8.26 | <0.001 | | 43.25 | | 9.51 | 44.32 | | 8.65 | 47.64 | | 8.75 | <0.001 | |
| TG (mg/dL) | 187.39 | 26.27 | 194.16 | | 26.51 | 217.48 | | 25.51 | <0.001 | | 208.61 | | 26.73 | 199.41 | | 27.35 | 185.82 | | 28.18 | <0.001 | |
| TC (mg/dL) | 182.35 | 18.39 | 182.34 | | 17.30 | 192.52 | | 17.33 | <0.001 | | 188.30 | | 18.68 | 185.86 | | 17.63 | 179.55 | | 17.39 | <0.001 | |
| SBP (mmHg) | 12.20 | 1.65 | 12.56 | | 1.67 | 13.36 | | 1.56 | <0.001 | | 12.94 | | 1.65 | 12.68 | | 1.68 | 12.37 | | 1.70 | <0.001 | |
| DBP (mmHg) | 8.09 | 1.09 | 8.26 | | 1.04 | 8.85 | | .97 | <0.001 | | 8.63 | | 1.07 | 8.34 | | 1.03 | 8.14 | | 1.11 | <0.001 | |
| ALT (IU/L) | 41.39 | 15.07 | 45.21 | | 15.26 | 59.70 | | 13.96 | <0.001 | | 52.94 | | 16.12 | 48.62 | | 15.32 | 41.36 | | 16.91 | <0.001 | |
| AST (IU/L) | 32.91 | 13.92 | 36.71 | | 14.06 | 50.41 | | 14.85 | <0.001 | | 45.06 | | 15.59 | 39.70 | | 14.57 | 32.32 | | 15.43 | <0.001 | |
| GGT (IU/L) | 27.28 | 13.39 | 26.91 | | 12.50 | 34.39 | | 12.97 | <0.001 | | 31.57 | | 13.20 | 29.18 | | 13.63 | 25.58 | | 11.70 | <0.001 | |

ALT, alanine aminotransferase; AST, Aspartate Aminotransferase; BMI, body mass index; FBS, Fasting Blood Sugar; GGT, gamma-glutamyl transferase; HDL, high-density lipoprotein; HOMA-IR, homeostasis model of insulin resistance; HOMA-β, Homeostatic model assessment of β cell function; LDL, low-density lipoprotein; TG, Triglyceride.

Table 7. Correlation of the quantitative characteristics of the participants with two dietary pattern

|  | Unhealthy Diet | | Healthy Diet |  |
| --- | --- | --- | --- | --- |
| Age (years) | | .427 | -.370 | |
| Weight (Kg) | | .415 | -.355 | |
| BMI | | .450 | -.390 | |
| Waist circumference (cm) | | .551 | -.399 | |
| FBS (mg/dL) | | .408 | -.330 | |
| Fast Insulin (μU/mL) | | .456 | -.386 | |
| 2h Glucose (mg/dL) | | .329 | -.269 | |
| 2h Insulin (µU/mL) | | .202 | -.210 | |
| HOMA_IR | | .148 | -.222 | |
| HOMA_B | | .162 | -.147 | |
| LDL (mg/dL) | | .313 | -.293 | |
| HDL (mg/dL) | | -.259 | .249 | |
| TG (mg/dL) | | .447 | -.351 | |
| TC (mg/dL) | | .262 | -.252 | |
| SBP (mmHg) | | .261 | -.162 | |
| DBP (mmHg) | | .312 | -.201 | |
| ALT (IU/L) | | .467 | -.345 | |
| AST (IU/L) | | .483 | -.376 | |
| GGT (IU/L) | | .245 | -.209 | |

ALT, alanine aminotransferase; AST, Aspartate Aminotransferase; BMI, body mass index; FBS, Fasting Blood Sugar; GGT, gamma-glutamyl transferase; HDL, high-density lipoprotein; HOMA-IR, homeostasis model of insulin resistance; HOMA-β, Homeostatic model assessment of β cell function; LDL, low-density lipoprotein; TG, Triglyceride.

**References:**

1 Bedogni, G. *et al.* The Fatty Liver Index: a simple and accurate predictor of hepatic steatosis in the general population. *BMC gastroenterology* **6**, 33 (2006).

2 Angulo, P. *et al.* The NAFLD fibrosis score: a noninvasive system that identifies liver fibrosis in patients with NAFLD. *Hepatology* **45**, 846-854 (2007).
